# Zonisamide effects on sleep problems and depressive symptoms in Parkinson’s disease

**DOI:** 10.1002/brb3.2026

**Published:** 2021-01-05

**Authors:** Keisuke Suzuki, Hiroaki Fujita, Takeo Matsubara, Yasuo Haruyama, Taro Kadowaki, Kei Funakoshi, Yuji Watanabe, Koichi Hirata

**Affiliations:** ^1^ Department of Neurology Dokkyo Medical University Mibu Japan; ^2^ Department of Public Health Dokkyo Medical University School of Medicine Mibu Japan

**Keywords:** depressive symptoms, motor symptoms, Parkinson’s disease, sleep disturbances, Zonisamide

## Abstract

**Background:**

We aimed to evaluate the effect of zonisamide (ZNS) on motor symptoms and nonmotor symptoms such as depressive symptoms and sleep problems in Parkinson’s disease (PD) patients with or without tremor.

**Methods:**

We conducted a 3‐month, open‐label study to assess the effects of ZNS on motor symptoms, depressive symptoms and sleep problems. Twenty levodopa‐treated PD patients with motor fluctuation completed the study. Patients received 25–50 mg/day of ZNS and were assessed for the Japanese version of the Movement Disorder Society Revision of the Unified PD Rating Scale (MDS‐UPDRS) parts I, III, and IV, PD Sleep Scale (PDSS)‐2, Beck depression inventory‐2 (BDI‐II), and PD Questionnaire (PDQ‐8) at baseline and after 1, 2 and 3 months of treatment. Patients were categorized into the tremor group and nontremor group to assess changes in clinical parameters.

**Results:**

At 3 months, the scores on the MDS‐UPDRS parts I, III and IV significantly improved and off‐time reduced compared to baseline. Additionally, the PDSS‐2 total score significantly decreased at 3 months. Although there were no significant differences in changes in UPDRS part I, III, or IV between the groups after ZNS treatment, the tremor group had significant improvements in PDSS‐2 at 3 months and BDI‐II at 1, 2 and 3 months compared with the nontremor group.

**Conclusion:**

We showed the beneficial effects of ZNS on motor symptoms and sleep problems in levodopa‐treated PD patients with motor fluctuation. ZNS may be more effective for several nonmotor symptoms in PD patients with tremor compared with those without tremor.

## Graphical abstract

In this 3‐month open‐label study, we showed the beneficial effects of zonisamide (ZNS) on motor symptoms and sleep problems in levodopa‐treated PD patients with motor fluctuation. ZNS was more effective for sleep disturbances and depressive symptoms in PD patients with tremor compared with those without tremor.

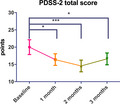

## 1 INTRODUCTION

Zonisamide (ZNS) has been licensed for treating motor symptoms in Parkinson’s disease (PD). Additionally, 50 mg/day ZNS reduced off‐time in a randomized double‐blind study (Murata et al., 2015). In a double‐blind, placebo‐controlled trial, ZNS reduced tremor amplitude in patients with essential tremor (Zesiewicz et al., 2007). In four of six patients (66.7%) with PD who had a history of postural or action tremor at least 5 years before the onset of PD, tremor (including action, postural and resting) improved after ZNS treatment (Bermejo, 2007). However, whether ZNS has differential effects in PD patients with and without tremors has not yet been studied.

Additionally, few studies have assessed the effects of ZNS on nonmotor symptoms (Bermejo et al., 2010). In this study, we aimed to evaluate the effect of ZNS on motor symptoms, depressive symptoms and sleep problems in patients with PD.

## 2 METHODS

We conducted a 3‐month, open‐label study to assess the effects of ZNS on motor symptoms, depressive symptoms and sleep problems. This study was performed in accordance with the Declaration of Helsinki and approved by the institutional review board of Dokkyo Medical University. All the participants provided written informed consent for participating in this study. From January 2018 to May 2019, 21 levodopa‐treated PD patients with wearing off symptoms (9 M/12 F; age 69.1 ± 8.1 years) were recruited. PD patients with psychosis or dementia, defined as scores of 20 or lower on the Mini‐Mental State Examination (MMSE), were excluded. At the beginning of the study, participants received 25 mg/day ZNS, and increasing the dose to 50 mg after 1 month was suggested. Other dopaminergic treatments were unchanged during this study. Except for one patient who was discontinued due to subjective feelings of lack of efficacy, 20 PD patients (8 M/12 F) completed this 3‐month study.

A PD diagnosis was made by board‐certified neurologists according to the UK Brain Bank Clinical Diagnostic Criteria (Hughes et al., 1992) and atypical parkinsonian syndrome, vascular parkinsonism or drug‐induced parkinsonism were carefully excluded by history taking, clinical examination and brain imaging studies. All patients were evaluated with Hoehn and Yahr (HY) staging and the Japanese version of the Movement Disorder Society revision of the Unified PD Rating Scale (MDS‐UPDRS) parts I (nonmotor experiences of daily living), III (motor examination) and IV (motor complications) at each visit (Kashihara et al., 2014). Off‐time was obtained from the MDS‐UPDRS part IV. For motor examination, the tremor score (subitems 3.15–3.17), rigidity score (subitems 3.3a–e), bradykinesia score (subitems 3.4–3.8 and 3.14) and axial score (subitems 3.1, 3.2, and 3.9–3.13) were obtained by summing the subitems of MDS‐UPDRS part III. Additionally, all the participants completed the PD Sleep Scale‐2 (PDSS‐2; Suzuki et al., 2012) to assess sleep problems, PD Questionnaire‐8 (PDQ‐8; Katsarou et al., 2004) to assess quality of life and Beck depression inventory (BDI)‐II to assess depressive symptoms at each visit. The levodopa equivalent dose (LED) was calculated for each patient (Tomlinson et al., 2010).

The patients were categorized into the tremor group and nontremor group, and changes in clinical parameters were compared between the groups. The tremor group was defined as a score of one point or greater on the MDS‐UPDRS part III subitems 3.15, 3.16, and 3.17.

### 2.1 Statistical analysis

Unpaired *t* tests and Fisher’s exact tests were employed for 2‐group comparisons where appropriate. The results for the clinical parameters including the MDS‐UPDRS parts I, III and IV, off‐time, PDSS‐2, BDI‐II, PDQ‐8 at baseline and after 1, 2 and 3 months were analyzed using repeated measures ANOVA followed by Bonferroni's multiple comparison tests. The clinical parameters between the tremor group and the nontremor group were compared at baseline and after 1, 2 and 3 months using two‐way repeated measures ANOVA followed by Bonferroni’s multiple comparison tests. We have analyzed effect size statistics generalized eta squared $\left(\eta_{G}^{2}\right)$ for two‐way repeated measures ANOVA. To evaluate a relationship between improvement in tremor and nonmotor symptoms, correlations between changes from baseline to 3 months in tremor score (sum of MDS‐UPDRS III items 3‐15–3.17) and in MDS‐UPDRS part I, PDSS‐2 and BDI‐II were evaluated by Spearman's rank correlation. Two‐tailed *p* values <.05 were considered statistically significant. GraphPad Prism for Mac (Version 8; GraphPad Software) was used for statistical analyses and figures.

## 3 RESULTS

**FIGURE 1:**
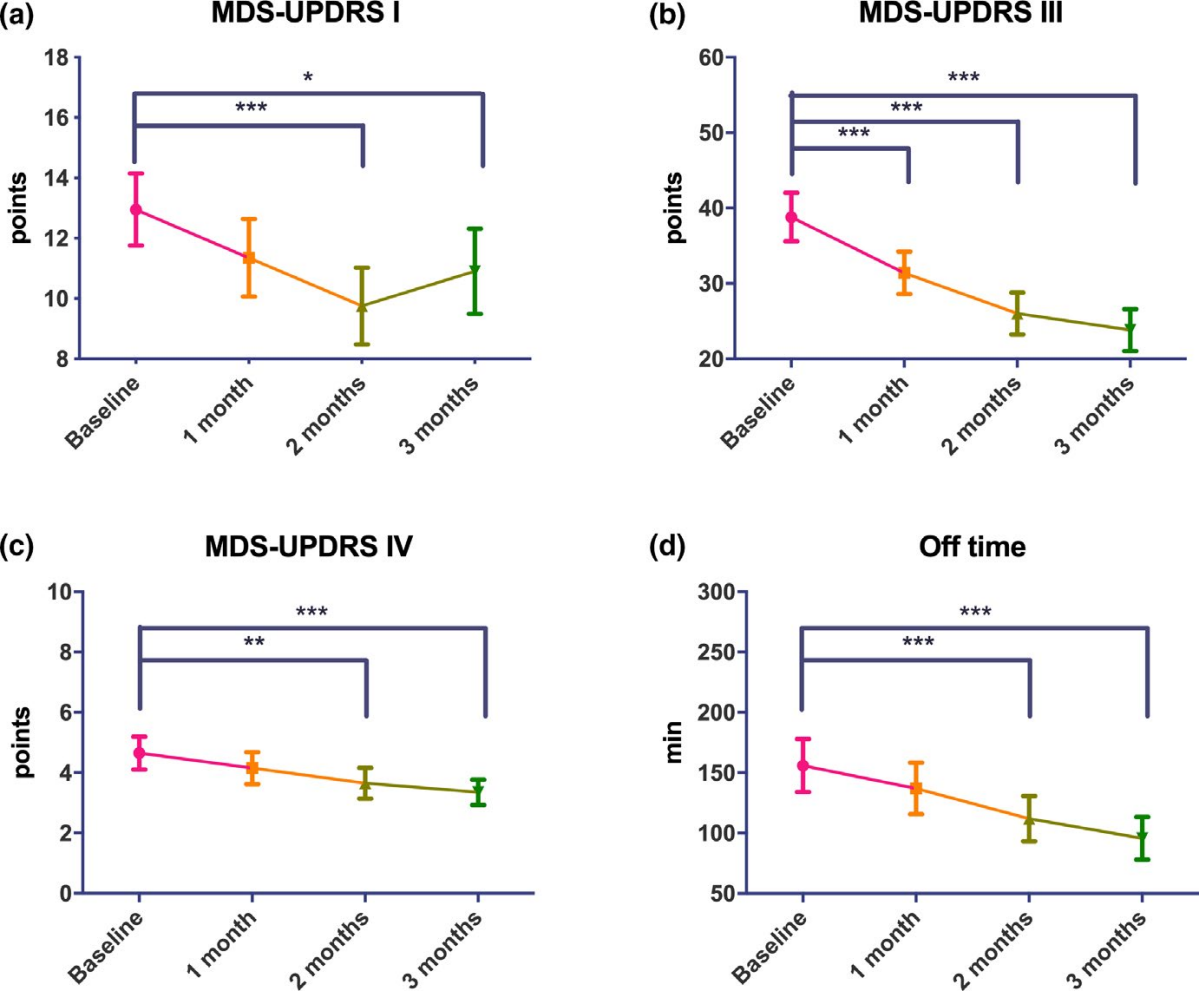
Changes in MDS‐UPDRS parts I, III and IV scores and off‐time after ZNS treatment. *p < .05; **p < .01; ***p < .001. Error bars represent standard errors of the mean

**FIGURE 2:**
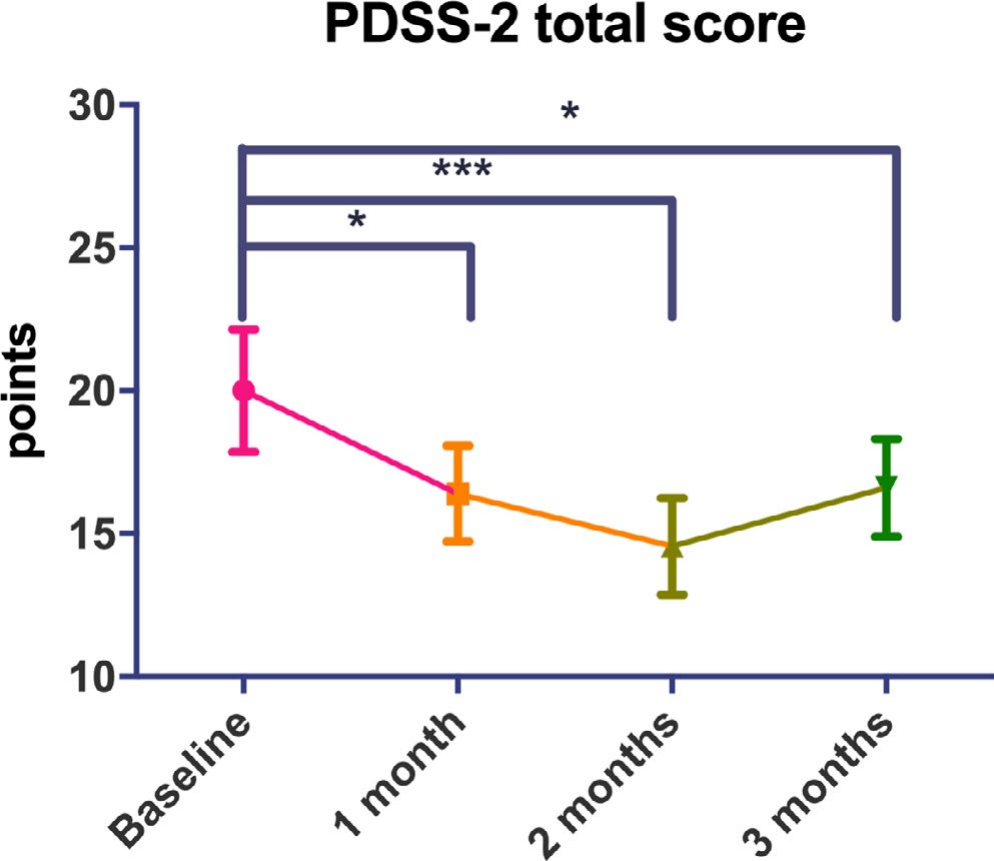
Changes in PDSS‐2 scores after ZNS treatment. *p < .05; ***p < .001. Error bars represent standard errors of the mean

Twelve (60%) and 8 (40%) were treated with 25 and 50 mg ZNS, respectively. The clinical background of the patients is shown in Table 1. There was no significant difference in the dose of ZNS between tremor and nontremor groups (37.5 ± 13.2 mg/day vs. 32.5 ± 12.1 mg/day, *p* = .38). The mean disease duration and HY stage were 5.6 ± 3.2 years and 2.6 ± 0.8, respectively. Repeated measures ANOVA followed by Bonferroni’s tests showed that at 3 months, the scores on the MDS‐UPDRS parts I (−2.1 points from baseline), III (−15.0 points from baseline) and IV (−1.3 points from baseline) significantly improved and off‐time reduced (−60.3 min from baseline) compared to baseline (Figure 1). The MDS‐UPDRS part III tremor, rigidity, bradykinesia and axial scores improved at 1, 2 and 3 months compared with baseline (Figure S1). The PDSS‐2 score also significantly decreased at 3 months (3.4 points from baseline; Figure 2). There was no significant difference in the BDI‐II or PDQ‐8 scores at any time point.

**TABLE 1:** Patient background

	PD (*n* = 20)	Tremor group (*n* = 10)	Nontremor group (*n* = 10)
Sex (M/F)	8/12	4/6	4/6
Age (years)	70.4 ± 5.8	69.2 ± 5.4	71.6 ± 6.3
Disease duration (years)	5.6 ± 3.2	5.8 ± 3.6	5.6 ± 3.0
Hoehn and Yahr stage	2.6 ± 0.8	2.6 ± 1.0	2.7 ± 0.7
MMSE	26.4 ± 2.5	25.9 ± 2.8	26.6 ± 2.4
Levodopa equivalent dose (mg/day)	469.1 ± 261.3	460 ± 255.3	478.1 ± 280.7
MDS‐UPDRS part I	13.0 ± 5.3	14.0 ± 5.8	11.9 ± 4.9
MDS‐UPDRS part III	38.8 ± 14.3	38.8 ± 14.3	38.8 ± 14.3
MDS‐UPDRS part IV	4.7 ± 2.5	3.1 ± 1.2	6.2 ± 2.4**
PDQ‐8 mean index	29.7 ± 22.1	43.4 ± 16.4	34.2 ± 10.8
PDSS‐2	20.0 ± 9.6	25.2 ± 9.4	14.8 ± 6.6*
BDI‐II	17.1 ± 8.6	20.6 ± 8.7	13.5 ± 7.1

Data are expressed as the mean ± *SD* (range)

BDI‐II, Beck depression inventory‐2; MDS‐UPDRS, Movement Disorder Society revision of the Unified PD Rating Scale; MMSE, Mini‐Mental State Examination; PDQ‐8, PD Questionnaire 8; PDSS‐2, PD Sleep Scale‐2.

* *p *< .05; **p < .01.

**FIGURE 3:**
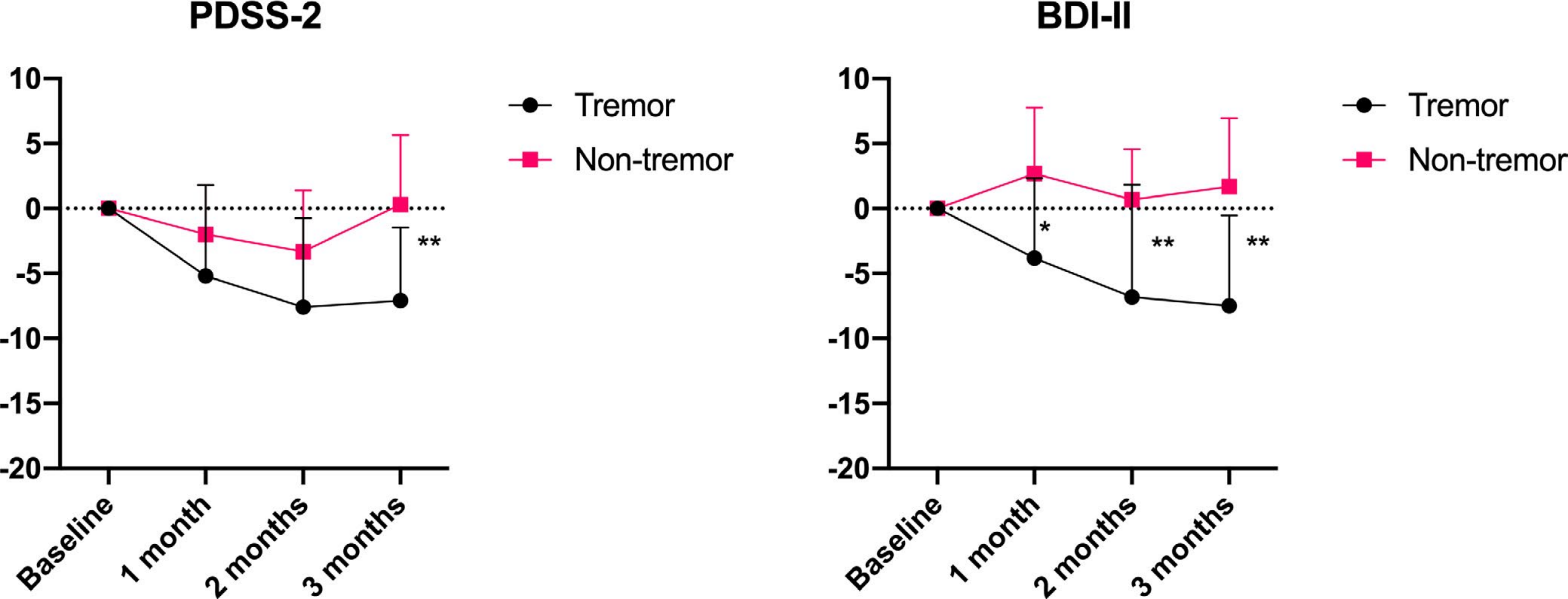
Changes in BDI‐II and PDSS‐2 scores between the tremor and nontremor groups. *p < .05; **p < .01. Error bars represent standard errors of the mean

The MDS‐UPDRS part IV score was lower and the PDSS‐2 score was higher in the tremor group than in the nontremor group. Other clinical factors, including the HY stage or MDS‐UPDRS part I or III score, were not significantly different between the tremor and nontremor groups. There were no significant differences in the main effects for group or time or interactions (group × time) for the MDS‐UPDRS part I, III or IV, off‐time or PDQ‐8 measures between the tremor and nontremor groups by 2‐way repeated measures ANOVA (data not shown). However, significant main effects for group (PDSS‐2, *F*(1, 18) = 4.961, *p* < .0001, $\eta_{G}^{2}$ = 0.21; BDI‐II, *F*(1, 18) = 10.24, *p* = .0050, $\eta_{G}^{2}$ = 0.01) and time (PDSS‐2, *F*(3, 54) = 8.518, *p* < .0001, $\eta_{G}^{2}$ = 0.08; BDI‐II, *F*(3, 54) = 3.059, *p* = .0359, $\eta_{G}^{2}$ = 0.02) and the interactions (PDSS‐2, *F*(3, 54) = 3.852, *p* = .0143, $\eta_{G}^{2}$ = 0.02; BDI‐II, *F*(3, 54) = 4.985, *p* = .0040, $\eta_{G}^{2}$ = 0.04) were shown for the PDSS‐2 and BDI‐II scores by 2‐way repeated measures ANOVA. Post hoc Bonferroni’s multiple comparison tests showed that the tremor group, compared with the nontremor group, had a significant difference in PDSS‐2 scores at 3 months (difference, 7.4 points) and BDI‐II scores at 1 month (difference, 6.5 points), 2 months (difference, 7.5 points) and 3 months (difference, 9.2 points) (Figure 3). We found a correlation between mean changes from baseline to 3 months in tremor score (sum of MDS‐UPDRS part III item 3.15–3.17) and in PDSS‐2 (*r* = 0.71, *p* < .001) and in BDI‐II (*r* = .71, *p* < .001) but not in MDS‐UPDRS part I (*r* = .35, *p* = .13).

## 4 DISCUSSION

In this 3‐month open‐label study, we showed the efficacy of ZNS on motor symptoms and motor complications using the latest version of the MDS‐UPDRS parts III and IV. Our results replicated previous findings showing the efficacy of ZNS on motor symptoms and motor complications using a previous version of the UPDRS parts III and IV (Maeda et al., 2015; Murata et al., 2007, 2015). Several parkinsonian motor features, such as tremor, rigidity, bradykinesia and axial symptoms, were assessed using the sum of several MDS‐UPDRS part III subitems, all of which significantly improved following ZNS treatment.

Previous studies had not assessed axial symptoms before and after ZNS therapy. Axial symptoms consist of postural instability, posture and freezing and are often unresponsive to conventional dopamine replacement therapy. However, we did show beneficial effects of ZNS on the axial score on the MDS‐UPDRS part III, presumably due to nondopaminergic mechanisms of ZNS (Suzuki et al., 1992; Yamamura et al., 2009). Although 60% of the patients received 25 mg ZNS and 40% received 50 mg ZNS in our study, the reduction in off‐time in our study at 3 months (60.3 min from baseline) was comparable to a previous randomized, double‐blind study of 422 PD patients, which found −0.436 and −0.719 hr reductions in off‐time at 3 months in the 25 and 50 mg ZNS groups, respectively (Murata et al., 2015).

One of the new findings from this study was that we found a significant reduction in the MDS‐UPDRS part I ‘‘nonmotor experiences of daily living” scores following ZNS therapy. There was no significant difference in UPDRS part I scores between baseline and 3 months after ZNS therapy in a previous study (Murata et al., 2015); however, the modified MDS‐UPDRS part I includes several new items, such as anxious mood, dopaminergic dysregulation syndrome, urinary problems, constipation and fatigue, compared to the UPDRS part I (Kashihara et al., 2014). Thus, the modification of MDS‐UPDRS part 1 by adding several nonmotor symptoms could have contributed to our study’s positive findings.

We failed to show significant differences following ZNS treatment in depressive symptoms and quality of life. However, we showed that ZNS treatment significantly improved sleep problems after 1, 2 and 3 months of ZNS treatment. ZNS has a long‐half life (−60 hr) and a low potential for interacting with other medications (Brodie et al., 2012) and has been reported to improve daytime motor and wearing off symptoms (Murata et al., 2007, 2015). Therefore, it is conceivable that it can improve nocturnal motor symptoms and nocturnal off symptoms. However, no previous studies had focused on whether PD‐related sleep problems change after ZNS treatment. In our study, the improvement in sleep problems assessed by the PDSS‐2, which address PD‐specific nocturnal problems, was likely due to improvements in nocturnal motor and partly nonmotor symptoms and nocturnal wearing off symptoms.

Next, we compared PD patients with and without tremor, as ZNS is known to improve tremor in patients with essential tremor (Zesiewicz et al., 2007) and those with PD (Maeda et al., 2015; Mochio et al., 2012). Activating dopamine synthesis and moderate inhibition of MAO‐B are thought be the main mechanisms for favorable effects of ZNS in PD (Murata et al., 2007). Mechanism for nonmotor symptoms is still unclear. However, antidepressant effects may be mediated via modulating dopaminergic and serotonergic function, and indirect reduction in glutamatergic activity and enhancing GABAergic activity (Ghaemi et al., 2006; Okada et al., 1998, 1999). Also, a pilot open trial showed efficacy of adjunctive ZNS on refractory anxiety and the authors suggested reduced excitatory neurotransmitters in the brain including amygdala may play a role (Kinrys et al., 2007). However, the effect of ZNS on motor and nonmotor symptoms between PD patients with and without tremor has never been compared. In this study, although there was no significant difference in changes in MDS‐UPDRS part I, III, or IV scores or off‐time between the tremor and nontremor groups after ZNS treatment, we found that the tremor group showed significant improvements in depressive symptoms and sleep problems compared with the nontremor group. We found that significant correlations between changes from baseline and end points in tremor score and PDSS‐2 and BDI‐II scores, supporting the effectiveness of ZNS on depressive symptoms and sleep problems was likely due to reduced tremor.

As a study limitation, we had no healthy control group, and the sample size of the PD patients was small. Second, off time was obtained by face‐to‐face interview by neurologists and a detailed on‐off fluctuation diary was not used. Third, it was not possible to assess whether motor and nonmotor symptoms return to baseline after interruption of ZNS treatment, as many patients continued to take ZNS after completion of this study. Further prospective large‐sample studies are needed to confirm our findings.

We showed the beneficial effects of ZNS on motor symptoms and sleep problems in levodopa‐treated PD patients with motor fluctuation. ZNS may be more effective for several nonmotor symptoms in PD patients with tremor than in those without tremor.

## CONFLICT OF INTEREST

Nothing to report.

## AUTHOR CONTRIBUTION

KS contributed to the study design, method, data acquisition and statistical analysis, and wrote the original manuscript. HF, TM, TK, KF and YW were involved in the study design, method and data acquisition. YH was involved in the study design, method, statistical analysis and review. KH contributed to the study design, review and supervision.

### Peer Review

The peer review history for this article is available at https://publons.com/publon/10.1002/brb3.2026.

## Supporting information

Figure S1

## Data Availability

The relevant data are within the paper, but the data sets from this study are available from the corresponding author on reasonable request.

## References

[brb32026-bib-0001] Bermejo, P. E. (2007). Zonisamide in patients with essential tremor and Parkinson's disease. Movement Disorders, 22(14), 2137–2138. 17853481 10.1002/mds.21717

[brb32026-bib-0002] Bermejo, P. E. , Ruiz‐Huete, C. , & Anciones, B. (2010). Zonisamide in managing impulse control disorders in Parkinson's disease. Journal of Neurology, 257(10), 1682–1685. 20509031 10.1007/s00415-010-5603-7

[brb32026-bib-0003] Brodie, M. J. , Ben‐Menachem, E. , Chouette, I. , & Giorgi, L. (2012). Zonisamide: Its pharmacology, efficacy and safety in clinical trials. Acta Neurologica Scandinavica. Supplementum, 194, 19–28. 10.1111/ane.12016 23106522

[brb32026-bib-0004] Ghaemi, S. N. , Zablotsky, B. , Filkowski, M. M. , Dunn, R. T. , Pardo, T. B. , Isenstein, E. , & Baldassano, C. F. (2006). An open prospective study of zonisamide in acute bipolar depression. Journal of Clinical Psychopharmacology, 26(4), 385–388. 16855456 10.1097/01.jcp.0000227702.72117.f5

[brb32026-bib-0005] Hughes, A. J. , Daniel, S. E. , Kilford, L. , & Lees, A. J. (1992). Accuracy of clinical diagnosis of idiopathic Parkinson's disease: A clinico‐pathological study of 100 cases. Journal of Neurology, Neurosurgery and Psychiatry, 55(3), 181–184. 10.1136/jnnp.55.3.181 PMC1014720 1564476

[brb32026-bib-0006] Kashihara, K. , Kondo, T. , Mizuno, Y. , Kikuchi, S. , Kuno, S. , Hasegawa, K. , Hattori, N. , Mochizuki, H. , Mori, H. , Murata, M. , Nomoto, M. , Takahashi, R. , Takeda, A. , Tsuboi, Y. , Ugawa, Y. , Yamanmoto, M. , Yokochi, F. , Yoshii, F. , Stebbins, G. T. …, MDS‐UPDRS Japanese Validation Study Group . (2014). Official Japanese Version of the Movement Disorder Society‐Unified Parkinson's Disease Rating Scale: validation against the original English version. Movement Disorders Clinical Practice, 1(3), 200–212. 25328906 10.1002/mdc3.12058 PMC4199098

[brb32026-bib-0007] Katsarou, Z. , Bostantjopoulou, S. , Peto, V. , Kafantari, A. , Apostolidou, E. , & Peitsidou, E. (2004). Assessing quality of life in Parkinson's disease: can a short‐form questionnaire be useful? Movement Disorders, 19(3), 308–312. 15022185 10.1002/mds.10678

[brb32026-bib-0008] Kinrys, G. , Vasconcelos e Sa, D. , & Nery, F. (2007). Adjunctive zonisamide for treatment refractory anxiety. International Journal of Clinical Practice, 61(6), 1050–1053. 17504366 10.1111/j.1742-1241.2007.01365.x

[brb32026-bib-0009] Maeda, T. , Takano, D. , Yamazaki, T. , Satoh, Y. , & Nagata, K. (2015). Zonisamide in the early stage of Parkinson's disease. Neurology and Clinical Neuroscience, 3(4), 127–130.

[brb32026-bib-0010] Mochio, S. , Sengoku, R. , Kono, Y. , Morita, M. , Mitsumura, H. , Takagi, S. , Kamiyama, T. , & Oka, H. (2012). Actigraphic study of tremor before and after treatment with zonisamide in patients with Parkinson's disease. Parkinsonism & Related Disorders, 18(7), 906–908. 22546334 10.1016/j.parkreldis.2012.04.007

[brb32026-bib-0011] Murata, M. , Hasegawa, K. , Kanazawa, I. , & Japan Zonisamide on PDSG . (2007). Zonisamide improves motor function in Parkinson disease: A randomized, double‐blind study. Neurology, 68(1), 45–50. 17200492 10.1212/01.wnl.0000250236.75053.16

[brb32026-bib-0012] Murata, M. , Hasegawa, K. , Kanazawa, I. , Fukasaka, J. , Kochi, K. , & Shimazu, R. (2015). Zonisamide improves wearing‐off in Parkinson's disease: A randomized, double‐blind study. Movement Disorders, 30(10), 1343–1350. 26094993 10.1002/mds.26286

[brb32026-bib-0013] Okada, M. , Hirano, T. , Kawata, Y. , Murakami, T. , Wada, K. , Mizuno, K. , Kondo, T. , & Kaneko, S. (1999). Biphasic effects of zonisamide on serotonergic system in rat hippocampus. Epilepsy Research, 34(2–3), 187–197. 10210034 10.1016/s0920-1211(98)00109-0

[brb32026-bib-0014] Okada, M. , Kawata, Y. , Mizuno, K. , Wada, K. , Kondo, T. , & Kaneko, S. (1998). Interaction between Ca2+, K+, carbamazepine and zonisamide on hippocampal extracellular glutamate monitored with a microdialysis electrode. British Journal of Pharmacology, 124(6), 1277–1285. 9720801 10.1038/sj.bjp.0701941 PMC1565497

[brb32026-bib-0015] Suzuki, K. , Miyamoto, M. , Miyamoto, T. , Tatsumoto, M. , Watanabe, Y. , Suzuki, S. , Iwanami, M. , Sada, T. , Kadowaki, T. , Numao, A. , Trenkwalder, C. , & Hirata, K. (2012). Nocturnal disturbances and restlessness in Parkinson's disease: Using the Japanese version of the Parkinson's disease sleep scale‐2. Journal of the Neurological Sciences, 318(1–2), 76–81. 22534309 10.1016/j.jns.2012.03.022

[brb32026-bib-0016] Suzuki, S. , Kawakami, K. , Nishimura, S. , Watanabe, Y. , Yagi, K. , Scino, M. , & Miyamoto, K. (1992). Zonisamide blocks T‐type calcium channel in cultured neurons of rat cerebral cortex. Epilepsy Research, 12(1), 21–27. 1326433 10.1016/0920-1211(92)90087-a

[brb32026-bib-0017] Tomlinson, C. L. , Stowe, R. , Patel, S. , Rick, C. , Gray, R. , & Clarke, C. E. (2010). Systematic review of levodopa dose equivalency reporting in Parkinson's disease. Movement Disorders, 25(15), 2649–2653. 21069833 10.1002/mds.23429

[brb32026-bib-0018] Yamamura, S. , Ohoyama, K. , Nagase, H. , & Okada, M. (2009). Zonisamide enhances delta receptor‐associated neurotransmitter release in striato‐pallidal pathway. Neuropharmacology, 57(3), 322–331. 19482038 10.1016/j.neuropharm.2009.05.005

[brb32026-bib-0019] Zesiewicz, T. A. , Ward, C. L. , Hauser, R. A. , Sanchez‐Ramos, J. , Staffetti, J. F. , & Sullivan, K. L. (2007). A double‐blind placebo‐controlled trial of zonisamide (zonegran) in the treatment of essential tremor. Movement Disorders, 22(2), 279–282. 17149715 10.1002/mds.21282

