# Supplementary material for: Zonisamide effects on sleep problems and depressive symptoms in Parkinson’s disease
Source: Brain Behav. 2021 Jan 5;11(3):e02026. doi: 10.1002/brb3.2026 (PMC7994695; doi:10.1002/brb3.2026)
Supplement: Supplementary file 1 — Figure S1 [file BRB3-11-e02026-s001.zip › brb32026-sup-0001-FigLegend.docx]

**Figure S1: Changes in MDS-UPDRS part III tremor, rigidity, bradykinesia and axial scores after ZNS treatment**

*p<0.05; **p<0.01; *** p<0.001

Error bars represent standard errors of the mean.
