# Supplementary figures and images for: Zonisamide effects on sleep problems and depressive symptoms in Parkinson’s disease
Source: Brain Behav. 2021 Jan 5;11(3):e02026. doi: 10.1002/brb3.2026 (PMC7994695; doi:10.1002/brb3.2026)

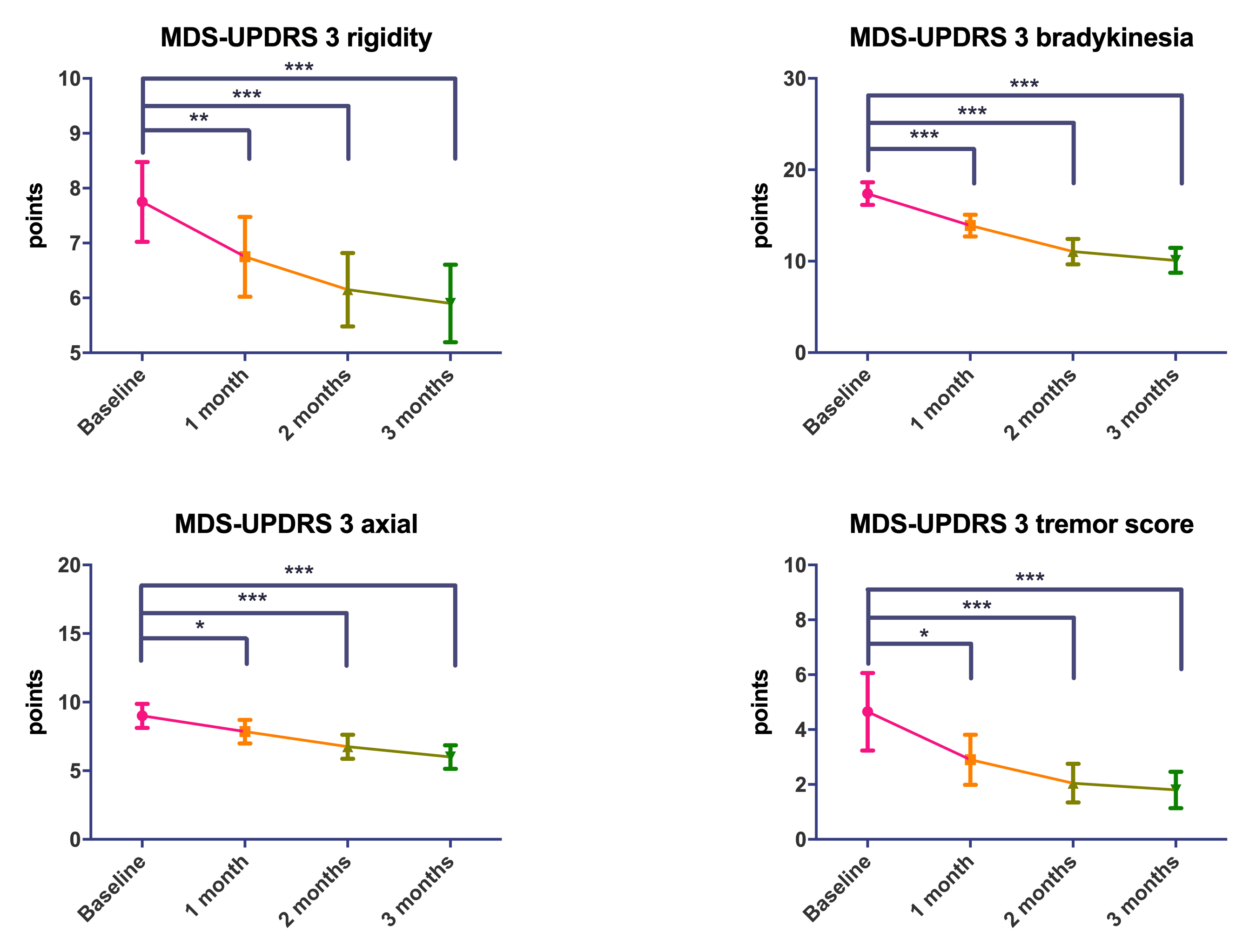

Supplement: Supplementary file 1 — Figure S1 [file BRB3-11-e02026-s001.zip › brb32026-sup-0001-FigS1.tiff]
